# New studies examine COVID‐19 risks among cancer patients

**DOI:** 10.3322/caac.21747

**Published:** 2022-07-07

**Authors:** Mike Fillon

“We believe everyone should do their part through vaccination and masking to protect this vulnerable community.”

—Changchuan (Charles) Jiang, MD, MPH

Two new studies examine COVID‐19 from the perspective of cancer survivors. One of the studies, which appears in the *Journal of the National Cancer Institute (JNCI)* (doi:10.1093/jnci/djab012), examines the prevalence among cancer survivors of medical factors associated with severe COVID‐19 disease. The other study, published in the *Journal of the National Comprehensive Cancer Network (JNCCN)* (doi:10.6004/jnccn.2021.7113), compares the incidence of adverse events among cancer survivors and persons without a history of cancer after SARS‐CoV‐2 vaccination.

Considered together, these 2 studies should help reassure cancer survivors regarding the safety of COVID19 vaccination and should alert them to their increased risk of severe outcomes from SARS‐CoV‐2 infection.

## Study Details and Results

For the JNCI study, researchers from the Roswell Park Comprehensive Cancer Center and the American Cancer Society used the 2016 to 2018 National Health Interview Survey to identify 6411 cancer survivors and 77,748 adults without a history of cancer in the United States. Excluded were nearly 3000 subjects with nonmelanoma skin cancer exclusively or those who were diagnosed with cancer before they turned 18 years old. The researchers noted the sociodemographic variables of the included subjects, including where they lived and their age, sex, race, ethnicity, educational level, insurance status, and personal economic levels.

The researchers found that 56.4% of the cancer survivors had 1 or more of the underlying risk factors that prior studies had shown to be associated with severe COVID‐19 disease, and 22.9% had at least 2. By contrast, only 41.6% of those without a cancer history reported at least 1 and only 10.8% reported 2 or more risk factors. Obesity ranked as the most common risk factor among cancer survivors (30.8%), followed by heart diseases (25.1%), diabetes (17.0), chronic obstructive pulmonary disease (9.2%), and chronic kidney disease 5.6%.

Among the cancer survivors, older subjects had greater prevalence of adverse medical conditions. In subjects aged 18 to 44 years, 47.6% of cancer survivors had at least 1 condition associated with severe COVID‐19 illness, and the prevalence increased to 51.6% and 61.2%, respectively, among cancer survivors aged 45 to 65 years and 66 to 84 years. A greater proportion of males than females had at least 1 risk factor (59.9% vs 54.0%, respectively). The prevalence of risk factors was also associated with education level, with at least 1 factor among 68.0% who had not completed high school versus 52.7% among those with education beyond high school. Having at least 1 risk factor was also more prevalent among Hispanics (61.4%) and non‐Hispanic Blacks (67.2) than non‐Hispanic Whites (55.2%). Risk factor prevalence was inversely related to family income (71.7% for those below the federal poverty level) and was highest among Southerners (59.2%) and Midwesterners (58.3%). Among individuals younger than 65 years, prevalence among persons with only public insurance (71.2%) was higher than among persons with private insurance (48.1%). The risk factor prevalence was highest among survivors of kidney, liver, and uterine cancers at 73.9%, 71.8%, and 71.5%, respectively. Sociodemographic characteristics influenced the prevalence of risk factors for severe COVID‐19 illness similarly among cancer survivors and cancer‐free control subjects, although prevalence percentages were higher among the former for all levels of sociodemographic characteristics.

**KEY ISSUES**

- The majority of safety and efficacy trials of the SARS‐CoV‐2 vaccines excluded patients with cancer, despite the greater risk these patients have to contract SARS‐CoV‐2.
- Differences in occurrence of vaccine side effects are very similar among cancer survivors and individuals without a cancer history.
- Medical conditions that are associated with severe COVID‐19 disease are more common among cancer survivors than among individuals without a cancer history.

In the JNCCN study, researchers noted that the majority of safety and efficacy trials of the SARS‐CoV‐2 vaccines excluded patients with cancer, despite the greater risk that these patients have to contract SARS‐CoV‐2 and become seriously ill. Between February 16, 2021, and May 15, 2021, they enrolled 2033 participants in a prospective, observational study conducted at the Fox Chase Cancer Center in Philadelphia, Pennsylvania. Each study participant received 2 doses of the Pfizer BNT162b2 vaccine, with the second doses administered 3 weeks after the first. There were 2 surveys given to the subjects. The first survey, asking about adverse reactions to dose 1 of the vaccine, was completed in person at the time of the second dose by 1752 patients. The second survey was completed either by telephone or online approximately 2 weeks after the second vaccine dose by 1260 participants. There were reports of COVID‐19 infection before vaccination by 3.4% of all respondents. Of the 1753 patients who completed at least 1 survey, 570 had no history of cancer and 1183 were cancer survivors, 211 of whom were receiving active cancer treatment (which included surgery, radiation and chemotherapy, immunotherapy, targeted therapy, or hormone therapy). Of the 1183 patients with a history of cancer, 92.5% reported that they had a solid malignancy and 7.5% had hematologic malignancies.

The cancer survivors tended to be older (with median ages of 68 vs 66 years) than the control group without any cancer history, and were more often male (42.2% vs 31.9%), and African American/Black (20.0% vs 9.8%.)

Symptoms occurred with similar frequency after vaccination of patients in both groups (73.3% with cancer vs 72.5% without cancer) with no significant differences after the first or second doses. The most common symptoms reported by patients with cancer after dose 2 included fatigue, joint pain, fever, chills, headache, and nausea.

The most common complaint among both subject groups was pain at the injection site. Injection site pain was reported slightly less often (but not significantly so) by cancer survivors than by control subjects (39.3% and 43.9%, respectively) for the first dose (*P* = .07) and 42.5% and 40.3% respectively, for the second dose (*P* = .45). Patients with cancer who were receiving active treatment reported significantly less injection site pain after dose 1 than patients with cancer who were not receiving active treatment (30.0% vs 41.4% respectively, *P* = .002). Cancer survivors reported generalized muscle pain somewhat more often that control subjects (16.5% vs 11.9%, respectively, *P* = .012), but this symptom lasted significantly longer in the latter group (mean, 2.2 vs 3.0 days; *P* =. 04). Other postvaccination symptoms such as joint pain, fever, chills, headache, and nausea did not vary significantly between cancer survivors and control subjects

## Study Interpretations

Lead author of the JNCI study, Changchuan (Charles) Jiang, MD, MPH, a clinical fellow in the hematology–medical oncology fellowship program at the Roswell Park Comprehensive Cancer Center in Buffalo, New York, says his research team believes that their study is important because it informs the public and guides policymakers on opportunities to prevent and control severe COVID‐19–associated illness through policies such as risk‐stratified vaccine distribution. “Specifically, our findings highlight the need to protect [cancer] survivors against COVID‐19 transmission in health care facilities and prioritize patients with cancer, cancer survivors, caregivers, and their health care providers in vaccination.”

Of particular concern is that cancer survivors are experiencing a steady increase in chronic disease burden, says Dr. Jiang. “The findings also remind us that cancer survivors are vulnerable [to severe COVID‐19 disease] due to many reasons that may not seem to be related to their cancer,” adds Dr. Jiang.

Coauthor of the JNCCN study, Eric M. Horwitz, M.D., professor and chair of the department of radiation oncology at the Fox Chase Cancer Center in Philadelphia, Pennsylvania, says he believes that his study is important because it focused on a group of people who are uniquely vulnerable to COVID‐19 and who benefit tremendously from being vaccinated. “Unfortunately, there is so much misinformation regarding vaccination and COVID‐19, and anything that can contribute to people getting vaccinated is important. There was and continues to be genuine concern regarding the effects of the vaccine on patients with cancer, and we hope that this study shows that the vaccine is well tolerated and that patients with cancer can get vaccinated just like other patients.”

Dr. Horwitz says that an important takeaway from his study is that patients with cancer need to believe that they are not at an increased risk for side effects compared to other patients, and that an important goal is to alleviate fear of vaccines. “I tell my patients that in addition to receiving their cancer treatment, one of the most important things they can do for their health is to get vaccinated.”

Dr. Jiang agrees. “Due to misinformation, we are seeing more COVID‐19 vaccine hesitancy over time and more unwillingness to follow the guidelines. Our study serves as a reminder that patients with cancer still need protection from COVID‐19 vaccination and herd immunity, especially when they have higher risks of severe COVID‐19 illness and may not respond as well to vaccination,” he says. “We believe everyone should do their part through vaccination and masking to protect this vulnerable community.

